# The development of methods to measure exposure to a major rabbit allergen (Ory c 1)

**DOI:** 10.3934/publichealth.2018.2.99

**Published:** 2018-04-09

**Authors:** Laura Willerton, Howard J Mason

**Affiliations:** 1 Public Health England, Manchester Royal Infirmary, Manchester, M13 9WZ UK; 2 Health & Safety Executive, Buxton, SK17 9JN UK

**Keywords:** rabbit allergens, aeroallergens, allergic diseases, air quality, environmental monitoring

## Abstract

Rabbits are used as laboratory animal models and are also popular domestic pets. Allergic responses to rabbit allergens have been documented in both settings, and several rabbit allergens identified. We have purified an 18 kD protein extracted from rabbit fur that was shown by N-terminal sequencing and mass spectrometry (MS) to be a lipocalin, identical to that identified as an odorant binding protein and an allergen with the formal nomenclature of Ory c 1. *De novo* sequencing of the MS peptide fragments gave additional primary sequence data of this protein. Polyclonal antisera were raised against the purified protein and used to develop two types of immunoassay. Ory c 1 content was measured in used rabbit bedding and household dust samples from homes keeping rabbits as pets. Atmospheric sampling was also undertaken in an animal facility undertaking rabbit experimental work. Ory c 1 levels in house dust where rabbits were kept as pets were between undetectable–41,290 ng·g^−1^, and in used bedding between 370–26,740 ng·g^−1^. Significantly higher house dust levels were found where rabbits spent large amounts, or all of, their time indoors. Personal air sampler levels within the animal facility were between 65–216 ng·m^−3^. Low levels (0.8–2 ng·m^−3^) were found in the facility's changing rooms, but undetected in the entrance lobby, office and laundry. We believe that these immunochemical assays may be used to identify activities in the occupational and domestic setting which produce higher levels of exposure to rabbit allergens, and where measures to control exposure may be warranted to reduce potential risk of allergic outcomes.

## 1. Introduction

A number of animal species that are used in medical/scientific research and also kept as household pets are a cause of allergic responses [1]. These species include mice, rats, hamsters, and rabbits. Sensitisation to rabbits and subsequent presentation of ocular, nasal and respiratory symptoms are well known from domestic [2],[3] and occupational exposure [4],[5].

In domestic exposure it has been shown that direct or even indirect rabbit contact in susceptible individuals may induce allergic sensitisation and that an increase in sensitisation may be expected with the increase in rabbit ownership [3]. In the domestic setting, atopy status and the degree of contact with rabbits are significant determinants of sensitisation to rabbits. Sensitisation to cats or dogs also increases the risk of developing allergy to other furry animals, such as rabbits [6]. An outpatient study in Naples of 753 subjects presenting with any positive skin prick test showed a prevalence of sensitisation to rabbit dander of 2.6% [3], with a corresponding prevalence of rabbit ownership in Naples of 1.6% [3].

The Pet Food Manufacturers' Association's UK survey in 2014–2015 suggested that 2.4% of households kept one or more rabbits as pets, equating to approximately 1 million UK domestic rabbits. Significant numbers of these domestic pets have access to, or live in, the house. A recent review specifically related to allergy and small furry pets noted their increasing popularity over the last 20 years, with up to 5% of US and European households keeping a small furry animal, but importantly noted that data on allergen levels in house dust are lacking [7].

Occupational exposure to rabbits is associated with the use of the animals in medical/scientific studies. In 2014 within the UK some 14,000 licensed procedures were carried out on rabbits out of a total of 1.93 million experimental animal procedures, the remainder largely involving mice [8]. Recent data on the prevalence of sensitisation to rabbits and symptoms in the occupational setting where rabbits are used as laboratory animals is lacking. A large epidemiological study of laboratory animal workers published in 1992 [5] stated that 30% of workers handling rabbits reported various symptoms. The proportion of workers developing symptoms during their first year of exposure to any species was highest for rabbits. A more recent study of those handling animals in a Japanese university reported a 11% serological prevalence of specific IgE to rabbits [9]. Some occupational exposure to rabbits, largely outside of the UK, may occur where they are farmed for human consumption or pelts/fur. A case study has even reported sensitisation and allergic symptoms in a magician employing a rabbit in his act [10].

The limited number of publications concerning the identification of rabbit allergens has suggested a number of major allergens, including 18 kD and 21 kD lipocalins [11]–[13]. The rabbit allergen with the nomenclature Ory c 1 is an 18 kD protein derived from saliva. It is likely that the salivary allergen is spread widely to the rabbits' fur by their grooming activity and after drying, allergen containing dust particles are spread to the wider area where the rabbit is active. Aerosolized allergens associated with rabbit activities or with human procedures such as handling the rabbits or cleaning-out, may lead to allergen levels likely to cause sensitisation or provoke symptoms in those already sensitised. Currently there are few data on the levels of the major identified rabbit allergens that can be encountered in either the domestic or occupational setting.

This paper describes the purification of an 18 kD protein found on rabbit fur, previously identified as a lipocalin allergen in rabbit saliva and named Ory c 1. The subsequent development of an immunoassay to this protein, capable of monitoring low levels that may be found in air samples, is described. We also report initial data on the levels of Ory c 1 found in bulk samples, such as used bedding from domestic pets and in household dust, together with airborne levels in facilities housing rabbits for scientific/medical purposes.

## 2. Materials and method

### 2.1. Allergen purification

Fur clippings were supplied from both a single lion head long-haired rabbit and a number of continental angora rabbits. Proteins were extracted by stirring the clipped fur samples at 10% w/v in PBS buffer with 2 mM PMSF for 2 hours. The extracted fur was squeezed and then filtered using a Whatman number 1 to remove any remaining solid material. The extract was then concentrated using an Amicon stirred cell incorporating a 3 kD membrane at 4°C. The concentrated extract was dialysed against water with stirring for 24 hours at 4°C, using 3.5 kD dialysis membrane (Spectrum) and subsequently lyophilised. The lyophilised material was re-dissolved in a small volume of 20 mM Tris pH 8 buffer and subject to exchange chromatography using a Resource Q 1 mL column on an AKTA purifier system (GE Healthcare, UK). The starting buffer was 20 mM pH 8 Tris buffer and a linear gradient was formed to 80% of 20 mM pH 8 Tris buffer containing 0.5 M NaCl as the gradient buffer. Protein elution was monitored at 280 and 210 nm and 1 mL fractions were collected for further analysis.

SDS gel electrophoresis was carried out at various stages of the extraction and purification process using pre-caste 10% XT MES gels in a mini-PROTEAN system (Biorad, UK). Gels were stained with either Coomassie G250 based dye (Gelcode blue, Thermo Scientific) or silver stained (Thermo Scientific). An identified, Coomassie stained 18 kD band in a collected peak from the ion exchange procedure was excised from the gel for protein identification and characterisation.

Protein concentrations were measured using a bicinchoninic acid assay standardised against purified bovine serum albumin.

Five milligrams of an 18 kD protein purified to greater than 95% was used to raise polyclonal antisera in two guinea pigs using a standard 56 day immunisation procedure (Envigo, UK). Antisera (coded 73 and 74) were purified to an Ig fraction using a standard protein a procedure (Thermo Scientific).

### 2.2. Protein identification and characterization

The excised 18 kD Coomassie stained band was subject to N terminal sequencing using an automated Edman degradation sequencer (AltaBiosciences, UK). Further stained gel slices of the same protein underwent protein identification (York University) using MALDI MS/MS mass spectrometry post trypsin digestion and a Mascot search (http://www.matrixscience.com/) of the NCBI and Uniprot databases. Subsequent *de novo* sequencing of fragmentation spectra of six parent ions was also carried out (York University) who also submitted the suggested *de novo* sequences to a MS-BLAST search using the on-line service provided by Sunyaev Lab at Harvard University.

### 2.3. Enzyme-linked immunosorbent assay

Aliquots of both protein A purified antisera from the two guinea pigs were biotinylated using 20 fold excess of SulfoNHS-LC-biotin (Vector labs) according to a standard method [14], dialysed against water to get rid of excess reagents and stored at −20°C in 50% glycerol at concentrations of approximately 1 mg/mL. A microtiter plate, non-competitive sandwich enzyme-linked immunosorbent assay (ELISA) was developed based on antisera coated wells, biotinylated antisera as detector antisera, avidin-poly-HRP (SDT Germany) as a signal amplification step and TMB for colour development. Identification of the appropriate antisera and optimisation of the concentrations of coating and biotinylated antibodies for maximum sensitivity were carried out using serial dilutions on a microtitre plate well coated at 2 µg.mL^−1^ with the 18 kD purified protein. Antisera, standards and samples were diluted in phosphate buffered saline (PBS) buffer containing 0.05% BSA and 0.05% Tween 20.

The cross reactivity of the ELISA was tested by running serial dilutions of pooled male urine from mice, rat, guinea pig and ferret, which are known to contain lipocalins that are major allergens [15] derived from animals routinely used as laboratory models. The initial total protein concentrations found in the urine of these respective species were 5.5, 12.9, 3.0, 7.4 mg·mL^−1^; serial 1/10 dilutions from an initial 1/100 dilution were analysed by the Ory c 1 ELISA.

### 2.4. Size-based quantitative immunoassay

Immunochemical staining of size-separated proteins in the range of 12–240 kD molecular weight were carried out using ProteinSimple's WES system (Biotechne, UK). The WES is an automated capillary-based system for performing size-based, immunochemical quantitation of proteins, using chemiluminescent detection. Antisera to Ory c 1 code 74 was used as the primary antisera at a dilution of 1/25. A secondary HRP labelled rabbit anti guinea pig immunoglobulin antisera (P0141, DAKO UK) was used at a dilution of 1/50. The optimised antisera dilutions for sensitivity and specificity were established through experimentation.

### 2.5. Samples from domestic and occupational settings

Samples of used bedding material were obtained from eight households keeping rabbits as domestic pets. Four of these households had rabbits that either lived as house pets or spent considerable amounts of time within the home. Samples of household dust from these premises, as well as from some control households, were collected using the Dust stream system (Indoor Biotechnologies). Each sample was obtained by vacuuming the upholstery of a chair or sofa in a living room for 5 minutes. The bedding and dust samples were frozen until analysis.

Air samples from an animal facility keeping rabbits were collected using air drawn through 1 µm PTFE filters (Millipore) in Institute of Occupational Medicine cassettes at 2 L·min^−1^. Both static and personal air samples were collected from the facility.

The stability of Ory c 1 collected on filters was investigated by spiking a number of 1 m PTFE filters (Millipore) with a known amount of purified protein in solution and drying in still air for 1 hour at 37°C. One third sections of the filters were stored at −20°C, 6°C and 22°C. At time points over a 40 day period, duplicate filters from each storage temperature were extracted and analysed for Ory c 1.

Domestic and occupational samples for analysis were extracted using 0.1% Tween 20 in PBS at 10% w/v for bulk samples and using 2 mL of the same buffer for extraction of filters from air sampling. Statistical analyses were performed using MedCalc Statistical Software version 17.9.7 (MedCalc Software bvba, Ostend, Belgium).

## 3. Results

### 3.1. Protein purification, identification & characterisation

Approximately 80–90 mg of total protein was initially extracted per 100 g of cut rabbit fur prior to any dialysis and concentration. After ion exchange chromatography, around 0.8–2.0 mg of the identified 18 kD protein was purified from 100 g of rabbit fur.

**Figure 1.**
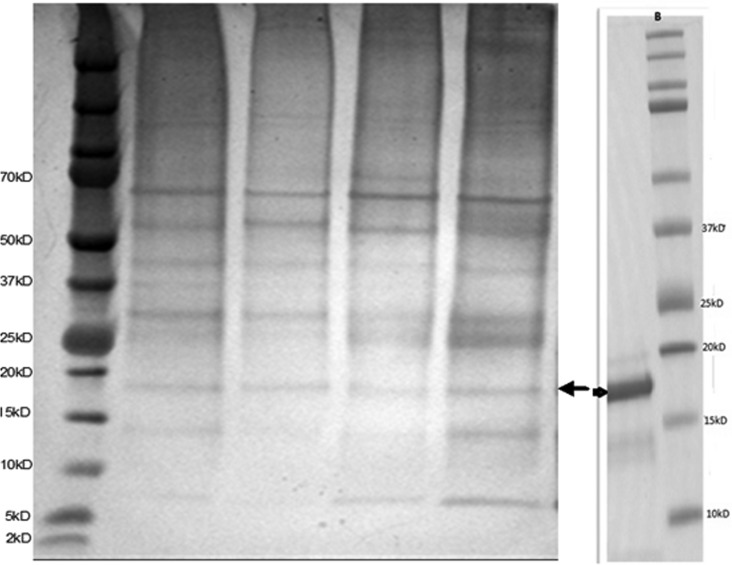
Reduced SDS gels of four initial rabbit extracts (gel on left) and purified 18 kD protein (gel on right).

A silver stained gel of the initial extract of rabbit fur from four different angora rabbits is shown in Figure 1 (left hand gel). A minor band at around 18 kD was visible in fur extracts from all four animals (arrow identifies); the relative intensity of this band on the gel is around 5–15% of total protein. This band was seen in extracts from all fur clippings donated.

Molecular weight markers (lane 1, left gel; lane 2 right gel) are shown in Figure 1. The arrows identify the 18 kD protein that was subsequently identified as Ory c 1 by N-terminal sequencing. The gel to the right [B] is a Coomassie stained gel showing the band that was excised for N-terminal sequencing.

**Figure 2.**
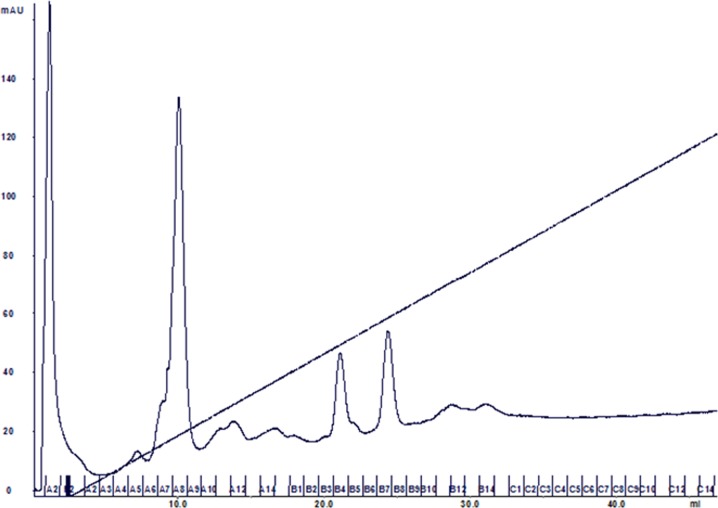
Ion exchange chromatogram of concentrated extract of clipped fur from rabbits.

The ion exchange chromatogram of the concentrated extracted protein is shown in Figure 2. The peak eluting in fraction B4 (elution 21–22 mLs, approximately 30% B buffer) contained the 18 kD protein by SDS gel electrophoresis (Figure 1; right hand gel). N-terminal sequencing of this excised band at 18 kD from the right hand gel in Figure 1 gave the following amino acid (AA) sequence:

**Table 1.** N-terminal sequencing.

AA number	1				5					10					15			18
Amino acid	V	D	P	A	Q	V	S	G	S	W	R?	T	A	A	I	A	S	D

This sequence of protein derived from the rabbit fur samples is identical to that published by Baker [13] for a rabbit salivary lipocalin protein with sequence similarity to an odorant binding protein, OBP-II (Uniprot) and was suggested to be the Ory c 1 rabbit allergen [13].

Protein identification using MALDI MS/MS mass spectrometry also identified the same protein using the NCBI database, again based on matching to the short peptide N terminal sequence that is the only sequence data for rabbit odorant binding protein II within the database. There was also a statistical match to donkey meiosis inhibitor protein. Further *de novo* sequencing suggested six further peptides sequences within the submitted rabbit protein on the gel slice, namely: VDPAQVSGSWR, FAALTEQDGIPR, VLQQDDGHIVFLNR, LLQQDDGHIVFLNR, TVVAQAWDGVYEAEFEGR, and DQVTHVTLAAGQDQELSDEDPSR. The first of these sequenced peptides is identical to the first eleven amino acids of the published N-terminal sequence for odorant binding protein (Ory c 1).

### 3.2. Immunoassays

The developed sandwich ELISA had a lower limit of detection of 0.29 ng·mL^−1^ with a standard curve ranging up to 12 ng·mL^−1^. Samples were measured after dilution 1 + 1 with assay buffer. The within batch coefficient of variation was 6.9% at 6 ng·mL^−1^.

The majority of the diluted preparations of mouse, rat, guinea pig and ferret urines were below the detection limit of the Ory c 1 ELISA. For rat, ferret and guinea pig male urine, the apparent measured Ory c 1 in these samples was less than 0.001% of the total urinary protein. For the male mouse urine, where the majority of urinary protein is lipocalin, the apparent Ory c 1 was around 0.1% of the total protein. These data suggest that issues of cross-reactivity from these species in the assay are unlikely to be a problem. The level of Ory c 1 measured in male rabbit urine was approximately 0.007% of the total urinary protein confirming that this protein, although a major allergen, is not a major urinary protein and may well be found in collected rabbit urine as a result of contamination.

The lower detection limit of the WES based system was approximately 50 ng·mL^−1^ and calibrated linearly up to 2000 ng·mL^−1^.

### 3.3. Domestic samples

Results from domestic setting are shown in Table 2. Analysis of used bedding material from households with rabbits as pets gave concentrations for the 18 kD protein (Ory c 1) of 370–26,740 ng·g^−1^ of straw.

Ory c 1 concentrations in household dust samples where rabbits were kept as pets were between ND and 41,290 ng·g^−1^ dust. Where the rabbits lived either as house pets or spent a significant amount of time within the house, the median settled dust ory c 1 concentration was 22,135 (range 608–41,290) ng·g^−1^ dust. This was significantly higher (*p* = 0.04; Mann Whitney test) than the median house dust level where the rabbits were living outside (median 280; range ND-756 ng·g^−1^ dust).

The Ory c 1 concentrations in dust samples from households not keeping rabbits as pets were below the limit of detection.

**Table 2.** Levels of Ory c 1 in samples of bedding and settled dust from households keeping rabbits as domestic pets. F = Female, M = Male.

Household	Rabbits, sex, neutered	Ory c 1 bedding ng·g^−1^	Ory c 1 house dust ng·g^−1^
*Animal(s) housed indoors*			
A, house rabbits (one and two rabbits housed separately)	1 × M + 1 × F Both neutered	1,783	33,830
	1 × F Neutered	2,952	
P, house rabbits	1 × M + 1 × F Both neutered	370	10,440
D, rabbit-housed outdoors but regularly indoors	1 × M Un-neutered	22,930	41,290
B, rabbit caged inside the home	1 × F Un-neutered	1,053	608
*Animal(s) housed outside the home*			
C, rabbit housed in garage	1 × M Un-neutered	26,740	ND
N, rabbit housed outside	1 × M Both neutered	3,530	560
T, rabbit housed outside	1 × M + 1 × F Both neutered	13,433	ND
J, rabbits housed outside	2 × M Both neutered	3,593	756
*Control samples*			
New, unused straw bedding	-	ND	-
G, control dust sample from house with no pets	-	-	ND
H, control dust sample from house with dog	-	-	ND
I, control dust sample from house with cat	-	-	ND

### 3.4. Occupational exposures

The atmospheric results from an animal facility using rabbits are shown in Table 3. The median of five personal atmospheric samples was 103 ng·m^−3^ (range 65–216 ng·m^−3^). The highest value was associated with “animal receipt”, an activity that involves significant handling of the animals. Matched static samples, where available, were lower than the personal samples. While no Ory c 1 was detected in an air sample taken from the entrance lobby, office and laundry, low levels were detectable in the male and female changing rooms (0.8 and 2 ng·m^−3^ respectively).

The Ory c 1 allergen seems to show considerable stability. From purified Ory c 1 applied to PTFE filters and employing the ELISA method, the allergen declined to 65% of the initial value over 40 days when the filters were stored at room temperature. When filters were stored refrigerated or frozen the loss was only about 10% over the same 40 day period.

**Table 3.** Individual atmospheric concentrations of Ory c 1 measured by ELISA in an animal facility using rabbits. ND = Non detected.

Sample No	Sample type	Air sampling (minutes)	Description of task or area monitored	ng·m^−3^
MA01	Personal	110	Rabbit room cleaning out	88
MA02	Static	207	Rabbit room	13
MA03	Personal	115	Rabbit procedure	105
MA07	Static	78	Animal receipt (Rabbits only)	47
MA08	Personal	73	Animal receipt	216
MA09	Personal	213	General duties	65
MA10	Personal	66	Cage washing	ND
MA11	Static	65	Cage wash	ND
MA12	Static	226	Male Changing Room	0.8
MA13	Static	229	Female Changing Room	1.9
MA18	Static	205	Entrance lobby	ND
MA19	Static	193	Dawn's office	ND
MA20	Personal	161	General duties	103
MA21	Static	229	Laundry room	ND
MA22	Static	218	Main Corridor	ND
MA23	Static	226	Office Room B61	ND

## 4. Discussion

We believe that this is the first publication describing both the immunochemical measurement of the major rabbit allergen Ory c 1, and also presenting some initial exposure measurements in both the domestic and occupational setting involving the species. Hilger [16] reported a polyclonal non-competitive ELISA to another rabbit allergen Ory c 3, and similarly to our study, found measureable allergen in dust from six households with pet rabbits but non-detectable in control households. No detection limit was reported for the assay and there were no measurements from occupational exposure to rabbits. Our measurement of Ory c 1 allergen levels in a small number of settled dust samples from households show that significant levels can be found, particularly where the animals either live, or spend a considerable amount of time indoors. However in comparison with UK published data [17] on major cat and dog allergen levels (Fel d 1, 109–385 µg·g^−1^ and Can f 1, 102–322 µg·g^−1^ respectively) in living room dust for houses with those pets, our results of 10–34 µg·g^−1^ of Ory c 1 for freely house accessing rabbits suggest lower levels of rabbit allergen in the dust reservoir. Allergen particles in settled house dust can readily be re-suspended into the atmosphere by many activities in the home, leading to a risk of sensitisation and asthmatic symptoms in those already sensitized. The value of 2 µg·g^−1^ of a major house dust mite allergen (Der p 1) in settled dust is often quoted as the threshold for the risk of sensitisation. Levels in house dust of 1–2 µg·g^−1^ have been suggested as thresholds for sensitisation for the major cat allergens (Fel d 1) and dog (Can f 1) respectively, with values greater that 8–10 µg·g^−1^ associated with allergic symptoms in those already sensitised. The previous lack of an assay to measure levels of rabbit allergen has precluded investigating if there is a similar relationship. However, rabbits have been shown to be the most prevalent cause of sensitisation from domestic animals after cats and dogs [18].

For most protein allergens, including those derived from mice and rats where occupational exposure monitoring has been extensive, dose-response relationships remain ill-defined. However, even where “safe” exposure levels have not been defined, monitoring has been shown to be helpful in identifying those relatively high exposure tasks where measures to control exposure, such as engineering systems or personal respiratory protective equipment, are likely to reduce risk [19]. Therefore a major aim of this work was to develop simple, practical immunochemical methods that could be used to monitor levels of a major rabbit allergen as a means of indicating and then controlling risk of exposure during various occupational activities.

Our atmospheric monitoring in a single animal facility using rabbits showed that airborne Ory c 1 allergen can be monitored in a manner that is already widely applied for occupational exposure to mice and rat allergens [19]. However, the rabbit dataset is currently too small to draw definitive conclusions about individual tasks or areas monitored. Previous published work on mice and rats has shown that static samples collected in the same areas where personal samples from staff were obtained, are generally lower, reflecting that proximity to laboratory animals, including handling of animals by staff, is an important determinant of airborne exposure to allergens [19]. It is noteworthy that whereas mice and rats in laboratory animal facilities are often housed in individually ventilated cages in racking, rabbits tend to be housed in floor cages.

Currently we have no information on the distribution of the rabbit allergen particle sizes. This will influence the extent of distribution of the allergen in the near environment, the likelihood of its wider dispersion and the penetration of allergen into the airways, modifying the nature and extent of symptoms in those already sensitised. The levels of contamination of bedding material suggests that, either in the domestic or occupational setting, the disposal of used bedding is a potential task that may liberate significant levels of airborne allergen.

Allergens in mammals appear to generally fall into three protein classes: Lipocalins, secretoglobilins and albumins [20]. We highlight that a protein with the same N-terminal sequence to a proven salivary lipocalin allergen [13] has been readily extracted from clipped rabbit fur, although given the grooming habits of rabbits this is not unexpected. Our stability data shows that this protein is relatively stable across a wide temperature range. The N-terminal sequencing of the purified 18 kD protein showed the glycine-x-tryptophan [G-X-W] triplet at amino acid positions 8–10 confirming the first conserved motif found in the lipocalin superfamily [21]. There are also three dimensional structural similarities between lipocalins [22]. Other lipocalin allergens include mus m 1 found in mice, rat n 1, Cav p 1 in guinea pig, Can f 1 from dogs, Bos d 2 from cattle and Equ c 1 from horses. A degree of cross-reactivity between epitopes of the various lipocalins of different species may explain the common finding of atopics with multiple sensitivities to small furry animals. However, our immunoassays, although polyclonal based, do not indicate significant cross-reactivity to lipocalins that are found in mouse, rat, guinea pig, or ferret urine, and may be used to specifically monitor exposure to the rabbit lipocalin allergen Ory c 1.

## 5. Conclusions

The paper demonstrates the ability to purify a major rabbit allergen relatively easily and measure levels of the allergen in air and dust samples by immunoassay. This will help in understanding the allergic response to both a laboratory animal and increasingly common domestic pet, and subsequently controlling the risk.
